# Incarceration of transplanted kidney through incisional hernia

**DOI:** 10.1093/jscr/rjab217

**Published:** 2021-06-16

**Authors:** Atta Nawabi, Derek Marlor, Christopher Camarata, Clay D King, Perwaiz Nawabi

**Affiliations:** The University of Kansas, Department of Surgery, Kansas City, KS, USA; The University of Kansas, Department of Surgery, Kansas City, KS, USA; The University of Kansas, Department of Surgery, Kansas City, KS, USA; The University of Kansas, Department of Surgery, Kansas City, KS, USA; The University of Kansas, Department of Surgery, Kansas City, KS, USA

## Abstract

Transplanted allograft kidney herniation through an incisional hernia resulting in incarceration is a rare condition with only one other similar case reported in the literature. The primary imaging modalities used to diagnose kidney herniation are graft ultrasound, abdominal computed tomography and abdominal magnetic resonance imaging [Sugi et al. (Imaging of renal transplant complications throughout the life of the allograft: comprehensive multimodality review. *Radiographics* 2019;**39**:1327-1355)]. Treatment should be based on patient’s symptoms. This case report highlights the initial presentation of hematuria in a 57-year-old male that eventually led to the diagnosis of a right-sided incarcerated grafted kidney through an incisional hernia. Subsequently, the patient underwent transplant nephrectomy.

## INTRODUCTION

Advancements in kidney transplantation and immunosuppressive therapies have made postoperative complications uncommon. The most common complications include acute or chronic rejection, poor wound healing given the use of immunosuppressive medications, delayed graft function, thrombosis of renal vessels, anastomotic leak, lymphocele and incisional hernia [1–3]. Some of these complications require early identification and operative intervention. The most common complication requiring surgical interventions are anastomotic leak, ureteral obstruction, hematoma, vascular thrombosis and incisional hernia [2–4]. Commonly, surgical complications initially may mimic medical problems such as drug toxicity or transplant rejection [5]. Incisional hernias following renal transplantation occur in 8–20% of cases, depending upon surgical technique, particularly based upon the location of incision made. The rate of incisional hernia following transplant through a lateral incision is 1–7%, with a mean of 3.2%, making it an uncommon complication [2, 6]. Independent risk factors for developing an incisional hernia following kidney transplant are: obesity (body mass index [BMI] > 30), female sex, concurrent abdominal wall hernias, history of smoking, duration of surgery and multiple explorations [2]. The most common presentation of a lateral incisional hernia is tenderness or pain and bulging mass of reducible tissue at the site of the incision. Less commonly, incisional hernias present with bowel obstruction. Incisional hernia following a kidney transplant can usually be diagnosed in an office or emergency department setting with a focused physical examination. If the physical examination is inconclusive, ultrasound or abdominal computed tomography (CT) is diagnosic [1, 3]. Elective operative indication for an incisional hernia should be left to the discretion of the surgeon. However, it is important to note that performing a repair done with synthetic, biologic or biosynthetic mesh may limit access to the iliac fossa in the future or place the patient at risk for mesh infection. Additional consideration must be taken when repairing a hernia with mesh, given the immunosuppressed state of transplanted patients. Emergent operative intervention is indicated in the case of incarcerated hernia contents. Upon review of the current literature, there was only one reported case of a transplanted kidney found to be incarcerated in an incisional hernia [7].

## CASE PRESENTATION

This is a 57-year-old male with end-stage renal disease due to post-streptococcal glomerulonephritis with a history of living related donor renal transplant in 1984 into his right lower quadrant. The allograft then developed transplant nephropathy and he began dialysis in 2015 and was relisted for red o kidney tx. In 2018, the patient received an HLA and ABO compatible kidney transplanted to his left iliac fossa. Subsequently, his original renal transplant became incarcerated through an incisional hernia, and a transplant nephrectomy was then performed.

The patient initially presented to our transplant clinic for evaluation and workup of hematuria and persistent, activity limiting, right lower quadrant pain, not associated with nausea or vomiting. Due to the development of multiple skin malignancies on Tacrolimus his immunosuppression regimen at the time included Everolimus and prednisone. On physical examination, he was noted to be moderately tender to palpation in the right lower quadrant over the previously transplanted kidney, with no obvious bulge.

**Figure 1:**
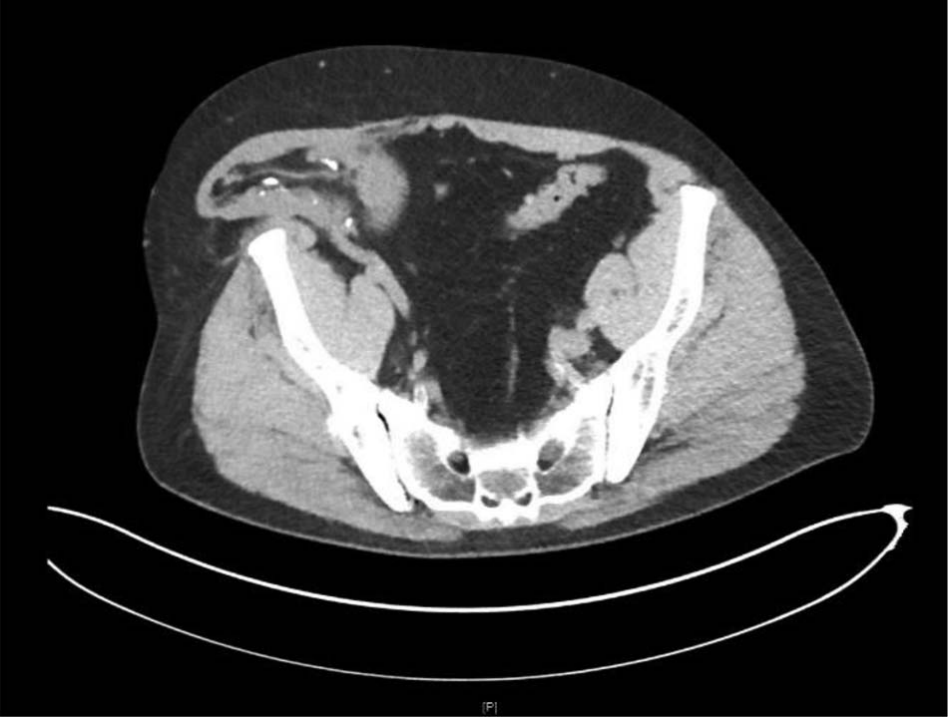
Axial view CT: herniation of the transplanted kidney through the right abdominal wall.

**Figure 2:**
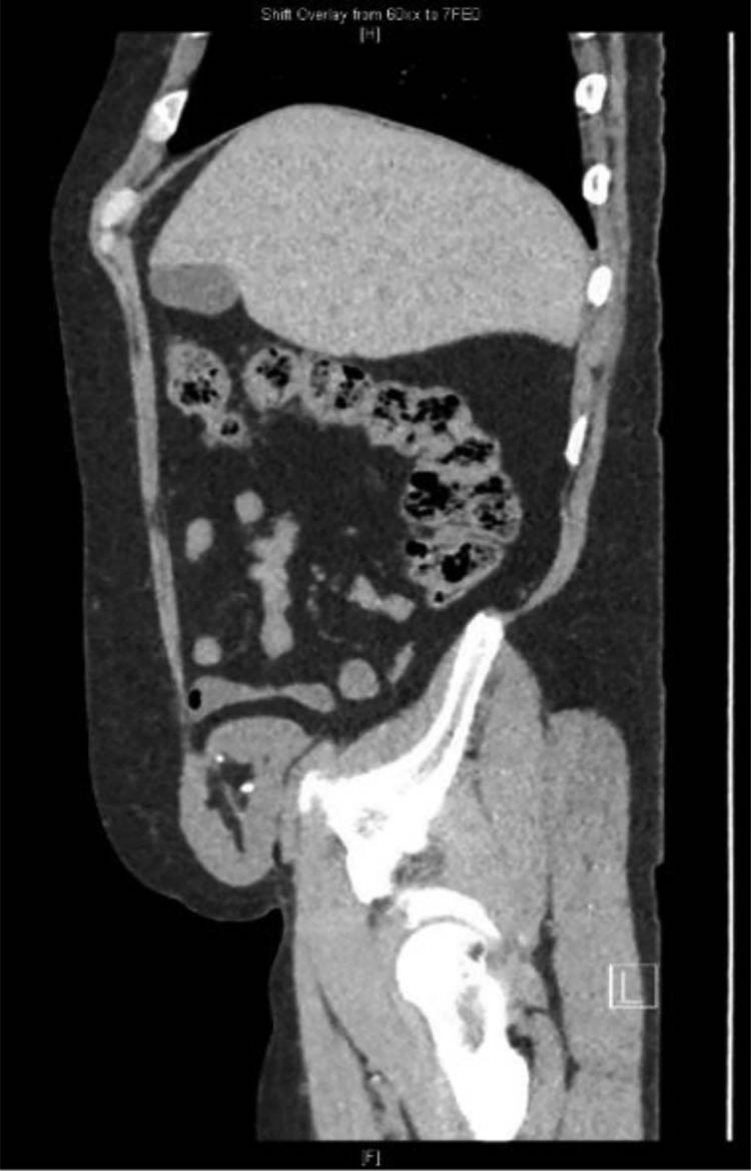
Sagittal view CT: herniation of the transplanted kidney through the right abdominal wall.

His workup was included but not limited to basic labs (CBC, CMP and UA), a renal transplant ultrasound, and a CT of the abdomen and pelvis. The CT scan revealed herniation of the right-sided donor kidney through an incisional hernia (Figs 1 and 2).

Given the patient's persistent, activity limiting pain, the decision was made to proceed to the operating room for right transplant nephrectomy and primary repair of incisional hernia.

**Figure 3:**
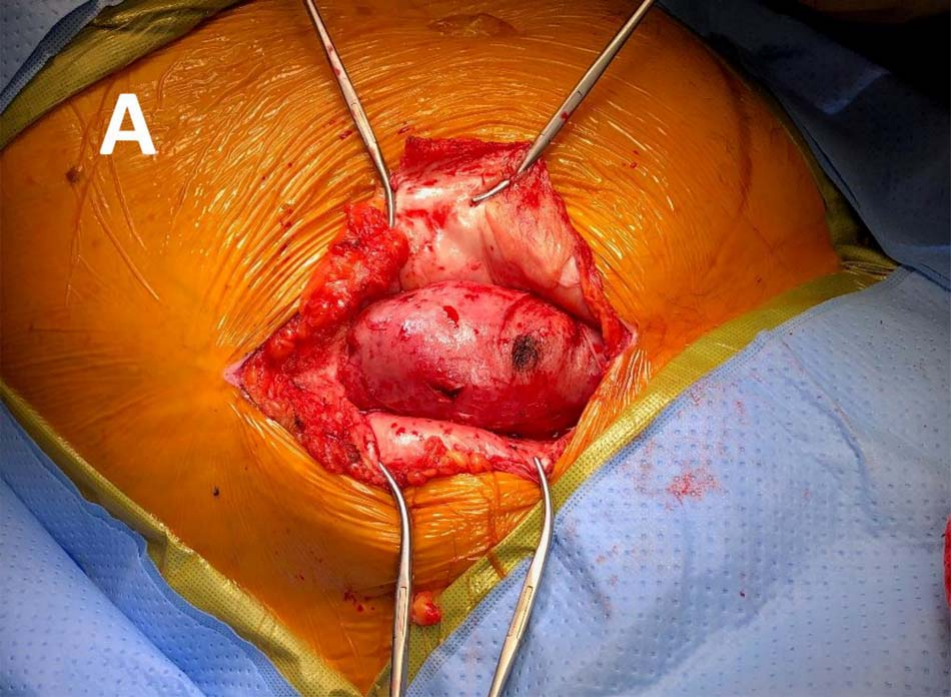
Intra operative photo revealing the herniated transplant kidney through the lateral abdominal wall fascia.

The nephrectomy was performed right lower quadrant, previously transplanted kidney scar. Dissection was carried down to the subcutaneous tissue where it was found that the transplanted kidney was incarcerated through the external oblique fascia (Fig. 3). The dissection continued and revealed a 3 × 3 cm fascial defect that resulted in the incarceration of the transplanted kidney. The fascia was then enlarged, and dissection was carried out into the retroperitoneum. The transplanted kidney renal vessels and ureter were exposed and stapled off above the patient external iliac vessels.

Given the small size of the fascial defect, and the patient being on Everolimus, the decision was made to repair the hernia primarily with 1-0 PDS suture in an interrupted fashion without tension. The wound was then closed in three layers using 2-0 absorbable sutures and the skin closed with staples.

The patient’s postoperative course was complicated by poor wound healing, subcutaneous seroma abscess that required drainage and antibiotics. His mTOR inhibitor was stopped to aid with wound healing and reduce risk of further complications.

## DISCUSSION

The rate of incisional hernia specifically following a transplant carried out through a lateral incision ranges from 1.1 to 7.0% with a mean of 3.2% [6]. Factors associated with incisional hernia were a BMI > 30, age > 50, cadaveric graft and reoperation through the same incision [6]. Additionally, smoking, lymphocele and partial closure in a single musculofascial layer are other risk factors [8]. Immunosuppressives such as mTORi (a class of drugs that inhibit the mechanistic Target Of Rapamycine) are known to carry increased risk of incisional hernia formation [5]. Our patient’s only associations with these independent risk factors were his age > 50 years and use of mTORi.

Intraoperatively, the decision to perform a hernia repair with mesh should be weighed against the risk of mesh infection and poor wound healing [9]. In this case we decided to repair the hernia defect primarily without tension because of the size of the hernia as well as the increased risk of poor wound healing and infection associated with mTORi.

This report emphasizes the importance of long-term follow up in transplanted patients. Prior to a transplant, it is important to consider independent risk factors to minimize complications [3]. In the postoperative course, providers should be thorough in gathering information to diagnose post-transplant complications. In the case of incisional hernias, a standard laboratory workup should always be obtained and imaging ordered beginning with transplant ultrasound followed by CT or magnetic resonance imaging if needed [1]. We hope the summary of current literature, data and imaging provided in this case report aids in diagnosing and treating patients with incarcerated allografted kidneys through incisional hernias.
